# Cost-effectiveness of cast treatment vs. surgery in elderly patients with substantially displaced intra-articular distal radius fractures: A trial-based economic evaluation

**DOI:** 10.1371/journal.pone.0339489

**Published:** 2026-01-30

**Authors:** Dirk P. ter Meulen, Johanna M. van Dongen, Ydo V. Kleinlugtenbelt, Gerald A. Kraan, J. Carel Goslings, Niels W.L. Schep, Nienke W. Willigenburg, Rudolf W. Poolman, On behalf of the DART study group

**Affiliations:** 1 Department of Orthopedic Surgery, OLVG, Amsterdam, The Netherlands; 2 Department of Health Sciences, Faculty of Science, Vrije Universiteit Amsterdam, Amsterdam Movement Sciences Research Institute, Amsterdam, The Netherlands; 3 Department of Orthopedic Surgery, Deventer Hospital, Deventer, The Netherlands; 4 Department of Orthopedic Surgery, Reinier de Graaf Hospital, Delft, The Netherlands; 5 Department of Trauma Surgery, OLVG, Amsterdam, The Netherlands; 6 Department of Trauma Surgery, Maasstad Hospital, Rotterdam, The Netherlands; 7 Department of Orthopedic Surgery, Leiden University Medical Center (LUMC), Leiden, The Netherlands; University of Pennsylvania Perelman School of Medicine, UNITED STATES OF AMERICA

## Abstract

**Background:**

The number of surgical procedures for distal radius fractures in the elderly has increased, even though most studies show little or no benefit over cast treatment. While medical costs of surgical treatment are higher than those of cast treatment, surgery may enable faster recovery and help patients maintain independence, potentially reducing their use of other healthcare resources and informal care.

**Methods:**

We evaluated whether cast treatment is cost-effective compared to surgery for patients aged 65 years or older with substantially displaced intra-articular distal radius fractures. A multicentre randomized controlled non-inferiority trial with an economic evaluation in 19 hospitals in the Netherlands. Participants completed (cost) questionnaires at baseline, 3, 6, 9 and 12 months after trauma. A total of 138 patients were randomized between cast treatment and surgery. Health-related quality of life was measured with the EQ-5D-3L; wrist function was measured with the Patient Rated Wrist Evaluation (PRWE). Costs were assessed from a societal perspective, including intervention costs, healthcare use, informal care, unpaid productivity losses, and patients’ own expenses.

**Results:**

The estimated difference in total societal costs was -€81 (95%CI -€3936 to €3773) in favour of cast treatment compared with surgical treatment. Compared with cast treatment, surgery resulted in improved wrist function (PRWE: −5.5; 95% CI: −10 to −0.7) and slightly higher QALYs (+0.039; 95%CI 0.012 to 0.066), equivalent to 14 days in perfect health. The incremental cost-effectiveness ratio was €15 per point improvement in PRWE and €2070 per QALY gained. Cast treatment’s probability of cost-effectiveness was low for all values of willingness to pay.

**Conclusion:**

Although cast treatment had lower direct costs, these were offset by higher informal and secondary care costs. Surgical treatment offered clinically relevant short-term benefits. From a societal perspective, surgery appears to be the more favorable option for elderly patients with displaced distal radius fractures.

## Introduction

Distal radius fractures in patients aged 65 years or older pose a significant burden on the healthcare system [1]. The rising incidence of such fractures, along with the aging population, necessitates a thorough understanding of the cost-effectiveness of treatment options. The management of patients with distal radius fractures typically involves either non-surgical cast treatment or surgical treatment, such as open reduction and internal fixation (ORIF). While ORIF allows for immediate mobilization and rehabilitation by creating a stable situation, it comes with an increased risk of complications and higher direct treatment costs [2]. Cast treatment, on the other hand, offers a non-invasive approach with lower complication rates and low direct treatment costs, but requires 4–6 weeks of wrist immobilization [3].

The number of surgical procedures in elderly patients has strongly increased in the past decades [4,5], even though most studies suggest that ORIF does not lead to a better patient-reported wrist function after a one year follow-up [6–11]. According to this literature, most elderly patients should therefore be treated non-surgically rather than surgically. This negates, however, the potential benefits of surgical treatment for elderly patients in terms of their ability to engage in activities, such as volunteering, taking care of their spouse, or maintaining independence at home. While elderly patients may typically not have paid employment, earlier return to daily activities may still contribute to their well-being and hence that of society as a whole. In addition, by optimally treating elderly patients, we might enable them to maintain an active and independent lifestyle, while avoiding indirect healthcare costs, such as physiotherapy, in-home care or informal care.

Economic evaluations comparing surgical treatment with cast treatment in younger populations show that surgical treatment is cost-effective, especially in patients with paid employment [12,13]. Two economic evaluations conducted among elderly patients showed that the cost-effectiveness of surgery compared with cast treatment is uncertain [14,15]. However, both these economic evaluations were conducted alongside clinical trials that did not show benefit from surgical treatment. Moreover, one of these economic analyses was solely performed from the hospital perspective. From a hospital perspective, surgical treatment can expected to be more expensive than cast treatment, because surgical treatment uses more resources, and without a relevant clinical benefit it will never be cost-effective. Therefore, we conducted an economic evaluation from a societal perspective alongside a non-inferiority, multicentre, randomized controlled trial (RCT) comparing surgical treatment (ORIF) with cast treatment for patients aged 65 years or older with a substantially displaced intra-articular distal radius fracture [16].

## Methods

### Design and participants

This is an economic evaluation from a societal perspective. This study was performed alongside the DART study [17] (Cast Versus Surgery for Displaced Intra-Articular Distal Radius Fractures in the Elderly a Randomized Clinical Noninferiority Trial). The DART study was a non-inferiority, multicentre randomized controlled trial (RCT) that compares the outcome of cast treatment with surgical treatment for intra-articular distal radius fractures in patients aged 65 years or older one year after trauma. Fracture characteristics had to be non-acceptable directly after reduction or due to re-displacement within 3 weeks post-trauma. Inclusion criteria for patients and the sample size calculation are listed in more detail in the DART study protocol [16]. The DART study was conducted in 19 hospitals in the Netherlands. The study was registered on clinicaltrials.gov (NCT03009890) and the Dutch Trial Registry (NTR6365). The study protocol has been published and contains a detailed description of the design and methods of the trial [6]. The study was approved by the Medical Research Ethics Committees United (MEC-U; NL56858.100.16).

### Randomization

Patients were randomly assigned to either cast treatment or surgical treatment. The age distribution between the treatment groups was balanced into two strata: 65–74 and 75 years or older. Mixed block randomization with block sizes of 4, 6, and 8 patients was employed for randomization. A web-based computerized randomization program was used to conceal treatment allocation.

### Intervention conditions

Patients received either cast treatment or surgical treatment. Patient in the cast treatment group received a below the elbow forearm cast for 4–6 weeks. Surgical treatment consisted of open reduction and internal fixation (ORIF). Both treatments were performed according to local protocols. Cross-over between groups was possible based upon the patient’s request or this decision could be made by their treating surgeon. The interventions are described in more detail in the DART protocol [16].

### Baseline characteristics

Baseline characteristics included standard patient characteristics (e.g., age, gender, and dominant side), baseline values of the clinical outcome scores and frailty scores [16].

### Clinical outcomes

Data for this economic evaluation were collected using questionnaires administered at baseline, 3 months, 6 months, 9 months, and 12 months after the trauma.

The primary outcome is the wrist function as reported by patients 12 months after trauma. Wrist function is measured with the patient rated wrist evaluation (PRWE) [18–20]. A higher score on the PRWE represents poorer wrist function, with 0 indicating perfect function and 100 indicating the worst possible function. A minimal clinically important difference of 14.0 was set as threshold for non-inferiority [21].

Health-related quality of life was measured using the EQ-5D-3L [22, 23]. The patients’ EQ-5D-3L health states were converted into utility scores using the Dutch tariff [24]. The number of QALYs gained during follow-up were calculated using linear interpolation between time points, with higher QALY values indicating a higher quantity and/or quality of life [25]. The minimal clinical important difference (MCID) for QALYs was assumed to be 0.074 [26].

### Cost outcomes

Costs were measured from a societal perspective, and included the costs of the intervention, other healthcare use, informal care, unpaid productivity losses, and patients’ own expenses. Costs of productivity losses from paid work were not included, because the population was ≥ 65 years at the time of trauma, and hence the overwhelming majority of them was expected not to have paid employment. Intervention costs were based on the cost of the treatment that they were allocated to (i.e., intention to treat), of which the unit costs were derived from hospital records (i.e., €627 for cast treatment; €5041 for ORIF). Other healthcare costs included costs related to the use of primary healthcare services (e.g., general practitioner, physical therapist) as well as secondary healthcare services (e.g., hospital stays). Healthcare costs were valued using standard prices derived from the “Dutch Manual for Costing Studies in Health Care”, and if unavailable, prices of professional organizations [27]. Informal care and unpaid productivity losses were assessed as the number of hours of care provided by family, friends, or other volunteers, and the number of hours that patients were unable to perform unpaid activities such as volunteer work, household tasks, or educational activities, respectively. Both were values using a recommended Dutch shadow price of €17.5/hour, which reflects the replacement cost of domestic help (i.e., the wage of a domestic cleaner). Information on the patients’ own expenses were derived from the patients. All costs were expressed in Euros 2023. Discounting of costs and effects was not necessary due to the follow-up of 12 months, as the short duration of the study eliminates the need to adjust costs and effects to their present value. The applied unit costs, price year, and data sources are presented in Table 1.

**Table 1:** Unit costs.

Cost category	Unit cost Euros (2023)	Unit	Source
**Intervention costs**			
Open reduction and internal fixation (ORIF)	5041	per procedure	Hospital records
Cast treatment	627	per treatment episode	Hospital records
**Primary care**			
General practitioner consultation	41	per contact	Dutch Manual for Costing (ZIN, 2014)
Physiotherapy session	41	per session	Dutch Manual for Costing (ZIN, 2014)
Home care	52	per hour	Dutch Manual for Costing (ZIN, 2014)
**Complications**			
Procedures for treating complications (e.g., hardware removal)	variable	per procedure	Hospital records
**Other secondary care**			
Outpatient visit (orthopaedic/trauma surgery)	113	per visit	Dutch Manual for Costing (ZIN, 2014)
Emergency department visit	323	per visit	Dutch Manual for Costing (ZIN, 2014)
Hospital day (rehabilitation center)	573	per day	Dutch Manual for Costing (ZIN, 2014)
Hospital day (surgical ward)	593	per day	Dutch Manual for Costing (ZIN, 2014)
**Informal care**			
Help by family members, friends, other volunteers	17.5	per hour	Dutch Manual for Costing (ZIN, 2014)
**Patient costs**			
Patient’s own expenses in relation to their fracture	variable	variable	Patient self-report
**Productivity costs**			
Unpaid productivity (e.g., voluntary work, schooling)	17.5	per hour	Dutch Manual for Costing (ZIN, 2014)

Abbreviation: ZIN = Zorginstituut Nederland.

Note: Costs were adjusted to 2023 euros using the consumer price index.

### Data analysis

In the main analysis, data were analysed by intention-to-treat. Data analyses were performed using the R codes developed by Jornada Ben et al. [28] Using “Multivariate Imputation by Chained Equations” and “Predictive Mean Matching” (PMM), missing data were multiply imputed, stratified by treatment group. This was done to ensure that imputations were generated within each treatment arm separately, thereby preserving potential differences between groups and preventing imputations from being influenced by data from the other arm. The imputation model included all available cost and effect data at the various measurement points, as well as several auxiliary variables (i.e., treating hospital, frailty, age, and grip strength) to increase the plausibility of the missing at random assumption (i.e., the assumptions that the probability of missingness depends on observed data, but not on the unobserved values themselves). For PMM, the 5 nearest neighbors were used (k = 5), meaning that for each missing value, five observed cases with the most similar predicted values were identified, and one of their actual observed values was randomly drawn to replace the missing value. Ten datasets were created in order for the loss-of-efficiency to be < 5% [29]. Each imputed dataset was analysed separately as specified below. Pooled estimates were calculated using Rubin’s rules, incorporating both within-imputation variability (i.e., uncertainty about the results from one imputed data set) and between-imputation variability (i.e., reflecting the uncertainty due to the missing information) [29]. The advantage of multiple imputation over other methods for handling missing data (e.g., complete-case analysis, regression imputation) is that it accounts for the uncertainty associated with the missing values by creating multiple plausible datasets, leading to less biased estimates and valid standard errors under the assumption of missing at random [30].

Seemingly unrelated regression (SUR) analyses were performed to estimate total cost and effect differences (i.e., ∆C and ∆E). An advantage of SUR, over—for example—ordinary least squares (OLS) regression, is that it accounts for the potential correlation between the error terms of the cost and effect equations, thereby providing more efficient and unbiased estimates of ∆C and ∆E[30]. These differences were corrected for baseline scores of the outcome as well as age. Incremental cost-effectiveness ratios (ICERs) were calculated by dividing the corrected differences in total costs by those in effects (i.e., ∆C/∆E). For QALYs, Incremental Net Benefits (INBs) were calculated using the Dutch willingness-to-pay thresholds for diseases with a low (€20,000/QALY), moderate (€50,000/QALY), and high disease burden (€80,000/QALY). INBs were not estimated for wrist function, as no established willingness-to-pay thresholds exist for this outcome. Bias corrected and accelerated (BCA) bootstrapping with 5000 replications was used to estimate the uncertainty surrounding the INBs, ICERs and cost differences. Bootstrapping allows for non-parametric estimation of uncertainty estimates without making strict distributional assumptions about the data. The advantage of BCA bootstrapping over other bootstrap methods is that it adjusts for both bias and skewness in the bootstrap distribution, resulting in more accurate and reliable uncertainty estimates, especially when the underlying data are not symmetrically distributed [30]. The uncertainty surrounding the ICER was graphically illustrated by plotting bootstrapped incremental cost-effect pairs (CE-pairs) on cost-effectiveness planes (CE-planes). A summary measure of the joint uncertainty of costs and effects was provided using cost-effectiveness acceptability curves (CEACs), which were estimated using the INB approach as described by Nixon et al. (2010)(INB = willingness-to-pay*∆E - ∆C) [31]. Such curves provide an indication of the probability of cast treatment being cost-effective compared with surgery at different values of willingness-to-pay (i.e., maximum amount of money decision-makers are willing to pay per unit of effect gained). As cost-effectiveness is based on superiority, we also estimated alternative CEACs, indicating the probability of cast treatment being non-inferior compared with surgery at different values of willingness-to-pay. For this, INBs were corrected for the MCID as follows: INB = willingness-to-pay*(∆E+MCID) - ∆C, where MCID was 14.0 and 0.074 for wrist function and QALYs, respectively. Analyses were performed in R studio according to the methods of Jornada Ben et al. (2023) [28]. Statistical significance was set at p < 0.05.

### Sensitivity analyses

Four sensitivity analyses were performed to assess the robustness of the results:

1. additionally adjusting for frailty, grip strength, and hospital;
2. applying the healthcare perspective, including only costs incurred within the formal Dutch healthcare sector;
3. conducting an *as-treated* analysis, defined as a switch between treatment arms within six weeks after trauma; and
4. applying two separate linear mixed models (one for costs and one for effects), each with a random intercept for hospital, bootstrapped jointly.

## Results

### Participants

Between January 23, 2017 and September 13, 2021, 138 participants were randomly assigned to cast treatment (n = 69) or surgery (n = 69). Four participants crossed over between groups (three from cast to surgery, one from surgery to cast at the patient’s request). Missing data were comparable between groups and showed a slight increase across time points. Per time point, approximately 4–20% of EQ-5D-3L and PRWE data and up to 22% of cost data were missing. In both groups, missingness was predominantly non-monotone (Appendix Table 1). Baseline characteristics of study participants are presented in Table 2.

**Table 2:** Baseline patient characteristics at randomization.

	Casting group		Surgical group	
	(N = 69)	N	(N = 69)	N
Age, Mean (SD)	75.7 (6.0)	69	75.6 (6.0)	69
Female, No. (%)	67 (97.1%)	69	61 (88.4%)	69
Dominant side injured, No. (%)	31 (48.4%)	64	28 (44.4%)	63
Diabetes mellitus, No. (%)	10 (16.1%)	62	8 (12.7%)	63
Corticosteroid use, No. (%)	4 (6.3%)	63	3 (4.8%)	63
Smoking, No. (%)	12 (19.0%)	63	4 (6.5%)	63
PRWE pre-trauma, Mean (SD)	2.69 (7.9)	58	1.47 (6.1)	55
DASH pre-trauma, Mean (SD)	4.2 (7.6)	58	2.9 (6.2)	55
EQ-5D-3L pre-trauma, Mean (SD)	0.92 (0.11)	58	0.94 (0.09)	55
Frailty score^a^, Mean (SD)	1.71 (1.60)	65	1.77 (1.96)	64
Frailty score^a^ ≥ 4, No. (%)	8 (12.3%)	65	11(17.2%)	64
Grip strength uninjured side^b^	38.5 (13.6)	66	44.8 (17.6)	64

Abbreviations: PRWE = patient rated wrist evaluation, DASH = disability of the arm shoulder and hand, EQ-5D-3L = EuroQol-5 Dimension 3 Level.

^a^Frailty measured with the Groningen Frailty Index (range 0–15, higher scores indicating more frailty).

^b^Measured in kilograms on the uninjured side.

### Clinical outcomes

Full details on the clinical outcomes have been reported in a separate publication and are summarized here to contextualize the economic findings. The trial was designed as a non-inferiority study with the PRWE at 12 months as the primary endpoint and a predefined non-inferiority margin of 14 points. In the primary intention-to-treat analysis, non-inferiority of cast treatment was not demonstrated: the between-group difference at 12 months was 6.0 points in favor of surgery, with a 95% confidence interval from −2.1 to 14.1 that slightly crossed the non-inferiority margin. In additional superiority analyses, PRWE scores were significantly better after surgery up to 9 months, with clinically relevant differences only up to 6 months. Recovery of grip strength and range of motion was faster in the surgical group during the first months after trauma. Quality of life (EQ-5D) showed small advantages for surgery in the early months, with no differences at 12 months. Complications and reinterventions were infrequent in both groups, with a higher rate of hardware removals after surgery [17].

### Economic evaluation outcomes

In this economic evaluation, for which missing data were multiply imputed, a mean difference in wrist function as measured by the PRWE of 5.5 (95%CI: 0.7 to 10.0) was observed between the cast treatment and surgery groups. For QALYs, the mean difference was –0.039 (95%CI: –0.066 to –0.012), indicating that the cast treatment group gained fewer QALYs during follow-up compared with the surgery group, equivalent to approximately 14 fewer days in perfect health (i.e., 0.039 × 365). These results also demonstrate that cast treatment was non-inferior to surgery for both QALYs and wrist function (PRWE), as the predefined non-inferiority margins (0.074 for QALYs and 14.0 for PRWE) were not included within the 95% confidence intervals (Table 3).

**Table 3:** Incremental cost-effectiveness results of cast treatment compared with surgery (surgery as reference).

Outcome	ΔC → Incremental costs (€) mean (95% CI)	ΔE → Incremental effects mean (95% CI)	ICER → Incremental cost-effectiveness ratio (€/unit)	Probability of cost-effectiveness at a WTP of €0/unit of effect	Probability of cost-effectiveness at a WTP of €20,000/QALY	Probability of cost-effectiveness at a WTP of €50,000/QALY	Probability of cost-effectiveness at a WTP of €80,000/QALY
Main analysis – PRWE	−81 (−3936–3773)	5.5 (0.7 to 10.0)	−15	0.52	–	–	–
Main analysis - QALYs	−81 (−3936–3773)	−0.039 (−0.066 to −0.012)	2070	0.52	0.37	0.21	0.12
Sensitivity analysis 1 – PRWE ^a^	−1053 (−4773–2667)	3.5 (−1.5 to 8.4)	−303	0.71	–	–	–
Sensitivity analysis 1 – QALYs ^a^	−1053 (−4773–2667)	−0.028 (−0.055 to −0.0001)	37322	0.71	0.60	0.43	0.31
Sensitivity analysis 2 – PRWE ^b^	−3109 (−5920 to −568)	5.5 (0.7 to 10)	−568	0.98	–	–	–
Sensitivity analysis 2 – QALYs ^b^	−3109 (−5920 to −568)	−0.039 (−0.066 to −0.012)	35	0.98	0.94	0.76	0.51
Sensitivity analysis 3 – PRWE ^c^	−1050 (−4895–2795)	4.5 (−0.3 to 9.5)	−229	0.70	–	–	–
Sensitivity analysis 3 – QALYs ^c^	−1050 (−4895–2795)	−0.035 (−0.062 to −0.008)	29808	0.70	0.58	0.38	0.24
Sensitivity analysis 4 – PRWE LMM	405 (−3505–4316)	5.3 (0.5 to 10.1)	77	0.42	–	–	–
Sensitivity analysis 4 – QALY LMM	405 (−3505–4316)	−0.038 (−0.064 to −0.012)	−10633	0.42	0.29	0.16	0.11

Abbreviations: PRWE = Patient-Rated Wrist Evaluation; QALY = Quality-adjusted life year; ICER = incremental cost-effectiveness ratio.

^a^Adjusted for frailty, grip strength, and hospital.

^b^Healthcare perspective.

^c^Per-protocol (as treated) analysis.

Negative incremental costs indicate lower costs for cast treatment.

### Cost outcomes

Intervention costs and total healthcare costs were significantly lower in the cast treatment group compared with the surgical treatment group, whereas informal care costs were significantly higher in the cast treatment group. Total societal costs were similar between both treatment groups; however, confidence intervals were large. An overview of all other disaggregate cost differences can be found in Table 4.

**Table 4:** Mean costs per cost category (€, per patient).

Cost category	Cast treatment, mean € (SEM)	Surgery, mean € (SEM)	ΔC unadjusted, € (95% CI)	C adjusted for baseline and age, € (95% CI)
Intervention costs	819 (109)	4977 (64)	−4157 (−4158 to −3981)	−4184 (−4355 to −4013)
Primary care costs	1333 (413)	795 (200)	538 (−148–1224)	350 (−276–976)
Complication costs	283 (130)	150 (46)	133 (−58–325)	102 (−80–285)
Other secondary care costs	1927 (956)	1281 (1008)	647 (−1975–3268)	622 (−4024–3656)
Informal care costs	6617 (1741)	3572 (489)	3046 (499–5593)	3014 (500–5527)
Patient costs	28 (14)	106 (61)	−78 (−171–14)	−77 (−168–14)
Unpaid productivity costs	925 (203)	681 (119)	245 (−97–586)	218 (−125–560)
**Total healthcare costs**	**4362 (1096)**	**7202 (1038)**	**−2340 (−5576 to −104)**	**−3109 (−5920 to −568)**
**Total societal costs**	**11933 (2113)**	**11561 (1191)**	**372 (−3464–4208)**	**−81 (−3936–3773)**

Abbreviations: SEM = standard error of the mean; CI = confidence interval; PRWE = Patient-Rated Wrist Evaluation; QALY = Quality-adjusted life year.

Incremental costs (ΔC) are expressed as cast treatment minus surgery; negative values indicate lower costs for cast treatment.

### Cost-effectiveness from the societal perspective

**Fig 1:**
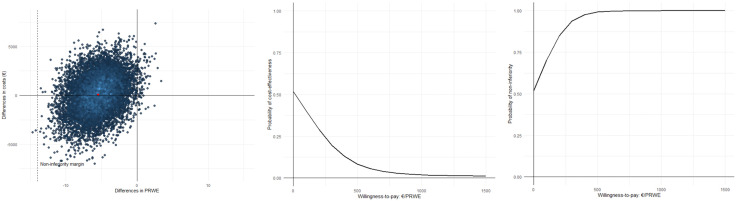
Cost-effectiveness analyses for PRWE (societal perspective). (a) Cost-effectiveness plane with incremental costs (€) on the y-axis and incremental effects (ΔPRWE) on the x-axis; dots represent bootstrap replications and the red dot the mean; the dashed line marks the non-inferiority margin (14 PRWE points). (b) Cost-effectiveness acceptability curve (CEAC) for superiority, showing the probability that cast treatment is cost-effective across willingness-to-pay thresholds. (c) CEAC for non-inferiority, showing the probability that cast treatment is non-inferior to surgery across willingness-to-pay thresholds.

The ICER for wrist function was −15, suggesting that cast treatment was – on average – associated with a societal cost saving of €15 per point increase on the PRWE compared with ORIF. This indicates that the cast treatment was – on average – “less costly” and “less effective” compared with ORIF, as a higher PRWE score indicates a poorer wrist function. The CEACs in Fig 1b-c show that if societal decision-makers are not willing to pay anything per point improvement in wrist function, the *probability of cast treatment being* ***cost-effective/non-inferior*** compared with ORIF was 0.57. It should be noted that the probabilities of cost-effectiveness and non-inferiority coincide when decision-makers are not willing to pay anything for additional units of effect, since both are entirely determined by the uncertainty surrounding the difference in costs. However, as cast treatment was – on average – “less effective” and cost-effectiveness is based on superiority, *the probability of* ***cost-effectiveness*** decreased with increasing values of willingness-to-pay, whereas the *probability of cast treatment being* ***non-inferior*** increased. The latter was due to the fact that only a very small proportion of the CE-pairs was located west of the non-inferiority margin (14 see Fig 1).

**Fig 2:**
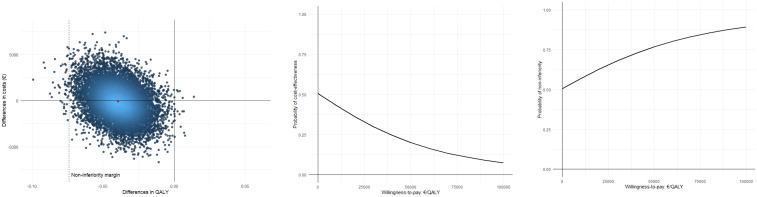
Cost-effectiveness analyses for QALYs (societal perspective). (a) Cost-effectiveness plane with incremental costs (€) on the y-axis and incremental effects (ΔQALYs) on the x-axis; dots represent bootstrap replications and the red dot the mean; the dashed line marks the non-inferiority margin (0.074 QALYs). (b) Cost-effectiveness acceptability curve (CEAC) for superiority, showing the probability that cast treatment is cost-effective across willingness-to-pay thresholds (€/QALY gained). (c) CEAC for non-inferiority, showing the probability that cast treatment is non-inferior to surgery across willingness-to-pay thresholds.

The ICER for QALYs was 2,070, indicating that cast treatment was, on average, “less costly” from the societal perspective, but also “less effective” than surgery. The INBs at the Dutch willingness-to-pay thresholds for diseases with a low (€20,000/QALY), moderate (€50,000/QALY), and high (€80,000/QALY) burden were €–676 (95%CI: –4792–3162), €–1,861 (95%CI: –6432–2391), and €–3,046 (95%CI: –8209–1792), respectively. The CEACs in Fig 2b-c show that if societal decision-makers are not willing to pay anything per QALY gained*, the probability of cast treatment being* ***cost-effective/non-inferior*** compared with surgery was 0.57. Again, *the probability of cost-****effectiveness*** decreased with increasing willingness-to-pay, while *the probability of* ***non-inferiority*** increased.

### Sensitivity analyses

Across all four sensitivity analyses, results were largely in line with the main findings. Sensitivity analyses 1 (additional adjustment), 2 (healthcare perspective), and 3 (per-protocol analysis) showed greater cost savings for cast treatment (€1053–€3109 versus €81 in the main analysis), indicating that cast treatment was generally “less costly” than surgery, although not all estimates reached statistical significance. Effect differences were similar to the main analyses, showing a small deterioration in wrist function (3.5–5.5 points) and a slight disadvantage in QALYs (–0.028 to –0.039). Sensitivity analysis 4, which applied separate linear mixed models with a random intercept for hospital and joint bootstrapping, yielded comparable results as well. Although cast treatment was estimated to be “more costly” rather than “less costly” in this analysis, the effect differences and probabilities of cost-effectiveness were similar to those observed in the main analyses.

## Discussion

From a societal perspective, the costs of cast treatment and surgical treatment in elderly were similar, showing that the considerably lower treatment costs of cast treatment were offset by higher primary and secondary healthcare, unpaid productivity, and informal care costs. Cast treatment’s probability of being cost-effective compared with surgical treatment for PRWE and QALYs was low for all values of willingness to pay, as were its probabilities of being non-inferior at reasonable values of willingness to pay. To illustrate, at the lower bound (i.e., 10,000/QALY) of the Dutch willingness to pay threshold for QALYs, cast treatment’s probability of being non-inferior was 0.61 (Fig 2c).

### Comparison with other studies

Hasselund et al. [11] conducted a randomized controlled trial with a similar patient population, specifically individuals aged 65 years or older who had unsatisfactory primary closed reduction or secondary displacement of distal radius fractures. In contrast to the DART trial, they concluded that cast treatment was non-inferior compared with surgical treatment after 12 months. In accordance with our results, however, they did observe that patients treated surgically had more favourable PRWE scores compared with patients in the cast treatment group, particularly at the short-term, although this difference did not reach clinical significance at 12-months follow-up. Alongside their trial, they also performed an economic evaluation from a societal perspective [14]. In contrast to our results, they found societal costs to be higher amongst surgically treated patients than those receiving cast treatment. This discrepancy may be explained by the substantial contribution of informal care costs in the cast-treatment arm of our study, a factor not considered in the analysis by Hasselund et al. Informal care refers to non-professional, unpaid assistance from family, friends, or other informal networks, including help with daily activities and occasionally medical tasks. The particularly high informal care costs observed in the cast-treatment group are consistent with the clinical results of the DART trial, which showed slower recovery of grip strength and range of motion after casting compared with surgery. This delayed recovery likely prolonged patients’ dependence on informal care. Facilitating access to professional home care or rehabilitation services could reduce reliance on informal care, although such services would also generate additional formal healthcare costs. [17].

Another similar trial was conducted by Lawson et al [10]. Their economic evaluation was conducted from a hospital perspective and found that surgical treatment was more expensive compared with cast treatment and that it was not associated with benefits in (short-term) clinical outcomes [15]. However, Lawson et al.’s adoption of the hospital perspective prevented cost savings in other categories from being picked up (e.g., in informal care costs), thereby inadvertently inflating cast treatment’s probability of being cost-effective compared with surgery.

The VIPER study by Mulders et al. [32] and the VIPAR study by Selles et al. [33] evaluated the cost-effectiveness of cast treatment versus surgery in a younger study population (18–75 year). In both studies patients were found to benefit from surgical intervention compared with cast treatment significantly. Although surgical treatment was more expensive from a hospital perspective, the younger, predominantly working-age population included in both studies experienced a delayed recovery with cast treatment compared to surgery, leading to productivity losses and hence, higher societal costs.

### Strengths and limitations

One of the biggest strengths of our economic evaluation is that it was conducted from a societal perspective. This perspective meant that we did not only assess the patients’ healthcare costs, but also assessed whether they were possibly offset by reductions in other cost categories (e.g., informal care, unpaid productivity costs). This is important as the healthcare costs of surgical interventions are typically higher than those of non-surgical ones, but could be offset by cost savings in other areas. Another strength is the use of state-of-the-art statistical methods for evaluating the intervention’s cost-effectiveness (i.e., the use of seemingly unrelated regression, bootstrapping, and multiple imputation) [34].

Limitations of this study include the use of self-reported data which may have caused recall bias (i.e., inaccuracies or distortions in participants’ memories due to factors such as forgetfulness or selective recall). Another concern is the low inclusion speed of the trial, which may be caused by a narrower range of patients than intended in our protocol, thereby compromising the generalizability.

Another issue with generalizability arose due to the exclusive reliance on data from the Dutch healthcare system and society. As evidenced by studies conducted by Hasselund et al. [14] in Norway and Howell et al. [15] in Australia, treatment costs exhibit significant variations across countries. Additionally, the degree to which individuals depend on informal care, a key determinant in the costs observed in our study, may vary significantly across different societies. Moreover, countries can differ extensively in terms of their sickness absence and social security policies. To illustrate, in the Netherlands, employees are generally entitled to up to two years of sick leave with wage replacement, whereas in other countries, such as the United States, compensation is often shorter in duration and less comprehensive. Such differences directly affect the valuation of productivity losses and can therefore lead to different cost-effectiveness outcomes. Hence, the current findings are most relevant to countries with comparable healthcare and social security systems (e.g., Germany, Belgium, or the Scandinavian countries), while caution is required when transferring them to settings with markedly different arrangements (e.g., the United States) [35,36]. The clinical DART study explored heterogeneity by age and frailty in exploratory analyses. This economic evaluation, however, did not include separate subgroup analyses by age or frailty.

In conclusion, from a societal perspective, cast treatment has similar costs compared with surgical treatment. This is mainly due to the higher informal care costs in the cast treatment arm. In addition patients seem to benefit from surgical treatment without experiencing more complications, especially in the short-term. Therefore, surgical treatment for elderly patients with displaced distal radius fractures appears to be a favourable option despite the relatively limited benefit after 12 months.
